# Endogenous retroviral ERVH48-1 promotes human urine cell reprogramming

**DOI:** 10.1186/s13619-024-00200-2

**Published:** 2024-09-13

**Authors:** Yuling Peng, Jieying Zhu, Qi Zhang, Ran Zhang, Zhenhua Wang, Zesen Ye, Ning Ma, Dajiang Qin, Duanqing Pei, Dongwei Li

**Affiliations:** 1 Key Laboratory of Biological Targeting Diagnosis, Therapy and Rehabilitation of Guangdong Higher Education Institutes, The Fifth Affiliated Hospital of Guangzhou Medical University, Guangzhou, 510799 China; 2 Bioland Laboratory (Guangzhou Regenerative Medicine and Health Guangdong Laboratory), Guangzhou, 510005 China; 3 CAS Key Laboratory of Regenerative Biology, Guangdong Provincial Key Laboratory of Stem Cell and Regenerative Medicine, Institutes of Biomedicine and Health, GIBH-HKU Guangdong-Hong Kong Stem Cell and Regenerative Medicine Research Centre, Hong Kong Institute of Science & Innovation, Guangzhou, Guangzhou, Guangdong 510530 China; 4 State Key Laboratory of Quality Research in Chinese Medicine, Institute of Chinese Medical Sciences, University of Macau, Macao, China; 5 Guangzhou National Laboratory, Guangzhou, China; 6 Laboratory of Cell Fate Control, School of Life Sciences, Westlake University, Hangzhou, 310024 China; 7 GuangDong Engineering Technology Research Center of Biological Targeting Diagnosis, Therapy and Rehabilitation, The Fifth Affiliated Hospital, Guangzhou Medical University, Guangzhou, China; 8 Guangdong Engineering Research Center of Early Clinical Trials of Biotechnology Drugs, The Fifth Affiliated Hospital, Guangzhou Medical University, Guangzhou, China

**Keywords:** Endogenous retroviruses, ERVH48-1, Human embryonic stem cells, Induced pluripotent stem cells, Urine cell integration-free reprogramming system

## Abstract

Endogenous retroviruses (ERVs), once thought to be mere remnants of ancient viral integrations in the mammalian genome, are now recognized for their critical roles in various physiological processes, including embryonic development, innate immunity, and tumorigenesis. Their impact on host organisms is significant driver of evolutionary changes, offering insight into evolutionary mechanisms. In our study, we explored the functionality of ERVs by examining single-cell transcriptomic profiles from human embryonic stem cells and urine cells. This led to the discovery of a unique ERVH48-1 expression pattern between these cell types. Additionally, somatic cell reprogramming efficacy was enhanced when ERVH48-1 was overexpressed in a urine cell-reprogramming system. Induced pluripotent stem cells (iPSCs) generated with ERVH48-1 overexpression recapitulated the traits of those produced by traditional reprogramming approaches, and the resulting iPSCs demonstrated the capability to differentiate into all three germ layers in vitro. Our research elucidated the role of ERVs in somatic cell reprogramming.

**Supplementary Information:**

The online version contains supplementary material available at 10.1186/s13619-024-00200-2.

## Background

Endogenous retroviruses (ERVs) are remnants of ancient retroviruses from the evolutionary process (Hayward et al. 2015). When exogenous viruses infect a host, they have the potential to integrate their genetic material into the host's genome, co-opting the host's ribosomal machinery to propagate. If this integration occurs in the host’s germ cells, the viral genetic material becomes permanently incorporated into the host genome, resulting in the formation of a proto-viral genome that is inherited following Mendelian inheritance patterns, ultimately giving rise to ERVs. (Waterston et al. 2002). Over evolutionary time, a substantial repertoire of ERV sequences has been conserved, constituting approximately 8% of the human genome today (Izsvak et al. 2016). Phylogenomic analyses suggest that ERVs likely originated from exogenous viral infections, becoming integrated and perpetuated within the genome during speciation and further disseminating through cross-species transmission events (Hayward et al. 2013).

ERVs typically span 7 to 11 kilobases and are characterized by a structural arrangement of 5' LTR-gag-pol-env-LTR 3' (Chen et al. 2024). The Long Terminal Repeat (LTR) regions regulate retroviral transcription and contain numerous transcription factor binding sites. The gag, pol, and env genes constitute their essential structural genes. Situated between the two LTR regions are pivotal binding sites: the Primer Binding Site (PBS), which engages with host cell tRNAs, and the Primer Primer Target (PPT), designated for primer binding during the synthesis of positive-stranded DNA (Vargiu et al. 2016). Throughout recombination and evolution, many ERVs have undergone genetic erosion, resulting in the absence of sequences within their internal coding domains; in some instances, only the LTR region, known as a solo-LTR, persists. A few recently integrated ERVs maintain an intact genomic architecture (Rowe and Trono 2011a). While the majority of ERVs have become non-functional, some retain the capacity to mobilize and proliferate extensively within the host genome, influencing the expression patterns of neighboring genes (Zhang et al. 2022). ERVs are not merely relics; they possess significant biological functions and are integral to processes such as embryonic development (Yang et al. 2022; Yu et al. 2022), immune system modulation (Zviran et al. 2019), and the determination of the host's phenotypic traits (Wang et al. 2023).

ERVs are expressed within embryonic cells and play a role in preserving pluripotency and stemness (Lu et al. 2014; Yu et al. 2022). During somatic cell reprogramming—whether in mice or humans—ERV expression patterns undergo significant shifts, predominantly characterized by substantial upregulation, which in turn influences the transcriptional activity of surrounding genes (Friedli et al. 2014). To date, the concept of ERVs as initiators within reprogramming systems remains uncharted territory.

Our recent work demonstrated that ERVH48-1, a protein-coding ERV, is expressed during the differentiation of hPSCs into cardiomyocytes, and that knocking out ERVH48-1 abolishes the cardiomyocyte cell fate decision (Zhang et al. 2024), indicating its role in cell fate regulation. We aimed to determine whether ERVH48-1 plays a role in the formation of pluripotency. Through the analysis of bulk RNA-seq and scRNA-seq data derived from human urine cells (UCs) and human pluripotent stem cells (hPSCs, H1) (Huang et al. 2021; Zhong et al. 2023; Abedini et al. 2021), we observed that ERVH48-1 was absent from urine cell samples yet robustly expressed within hPSCs (H1). This particular endogenous retrovirus (ERV), found exclusively in primate, is one of the more recently integrated elements within the evolutionary timeline of the genome. ERVH48-1 has been shown to be actively expressed and distributed at the critical maternal–fetal interface during early embryonic development, where it plays a role in preventing trophoblast fusion events induced by ERWW-1 through a competitive inhibition mechanism (Sugimoto et al. 2021). To explore the influence of ERVH48-1 on the reprogramming of pluripotency, we over expressed ERVH48-1 into a human urine cell reprogramming system, employing non-integrating in vitro chromosomal vectors (Wang et al. 2013). Our results demonstrated that ERVH48-1 significantly enhanced the reprogramming process by activate the pluripotency network, resulting in induced pluripotent stem cells (iPSCs) with phenotypic, qualitative, and differentiation profiles comparable to those of conventionally derived iPSC clones. Collectively, our research has shed light on the previously unrecognized contribution of ERVH48-1 to the intricate process of cellular fate reprogramming.

## Results

### Transcriptome sequencing analysis reveals ERVH48-1 expression in human embryonic stem cells

**Fig. 1:**
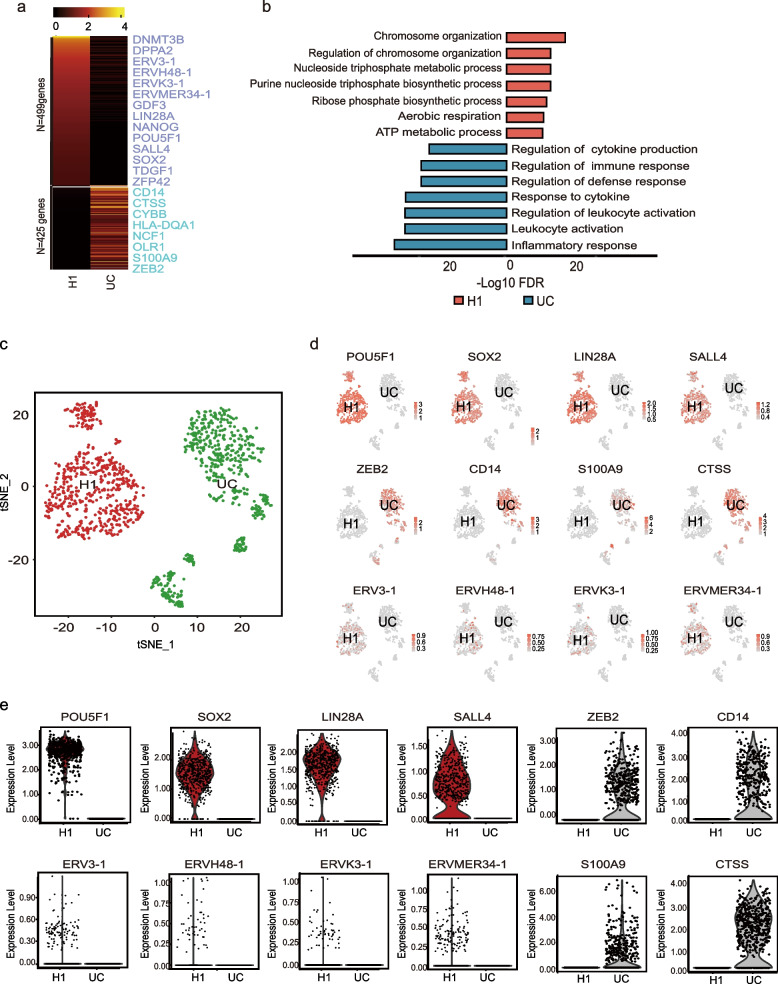
Transcriptome sequencing analysis reveals the expression of ERVH48-1 in hPSCs. **a** Heat maps of RNA-seq analysis results for UCs and H1 (hPSCs) showing 499 highly expressed genes in H1 and 425 in UCs. **b** Gene ontology analysis of UCs and H1. **c** Cluster analysis diagram of UCs and H1. **d** Genes with high expression in UCs and H1 were identified from the cluster, revealing ERV series expression in H1 but not in UCs. **e** Violin plot of the screened genes

To investigate the expression of ERVs in human embryonic stem cells, we analyzed bulk RNA-seq data from human pluripotent stem cells (H1) and Urine cells (UCs) (GSE168392) (Huang et al. 2021). Genes with a Log2 (Fold Change) >  = 1 were selected for further analysis, revealing 499 highly expressed genes in H1 and 425 in UCs (Fig. 1a). Gene Ontology (GO) analysis indicated that H1 and UCs are associated with different biological processes (Fig. 1b). Next, we examined hPSCs single cell RNA-seq data (GSA: HRA003227) (Zhong et al. 2023) and urine cells (UCs) (GSE157640) (Abedini et al. 2021). Cluster analyses highlighted differences in gene expression between the two cell types (Fig. 1c-e). Notably, ERVH48-1 was expressed in H1 but not in UCs, suggesting that ERVH48-1 may play a role in regulates pluripotency and influence the reprogramming of UCs into human induced pluripotent stem cells (iPSCs).

### ERVH48-1 promotes the reprogramming of UCs to iPSCs

**Fig. 2:**
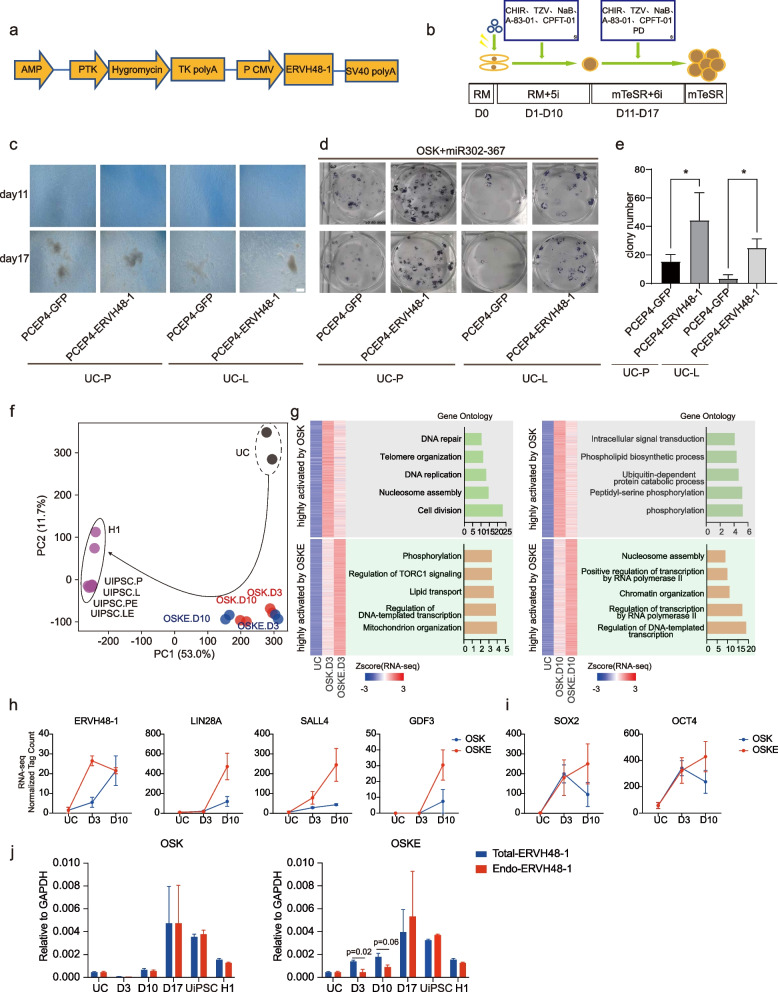
ERVH48-1 overexpression promotes the reprogramming of UCs to iPSCs. **a** Construction map of the ERVH48-1 overexpression vector: pCEP4-ERVH48-1. **b** UiPSCs acquisition protocol: the reprogramming-related plasmids were electroporated into UCs, RM plus 5i medium was used during reprogramming, RM plus 6i medium was used during proliferation, and mTeSR medium was used during purification. **c** Morphological changes of cells at different time points during the acquisition of UiPSCs from UCs of two healthy volunteers (scale: 500 μm). **d** AP staining of UiPSCs from two healthy volunteers. **e** Number of AP + clones of UiPSCs in each group. Data were from three independent experiments and are shown as the mean ± SEM. *P < 0.05, unmatched two-tailed T-test. **f** PCA analysis of the bulk RNA-seq data during reprogramming of UCs into iPSCs with or without ERVH48-1. OSK: reprograming with pCEP4-OSK + miR302-367 + GFP; OSKE: reprogramming with pCEP4-OSK + miR302-367 + ERVH48-1. D3: day 3; D10: day 10. **g** Heatmap showing differentially expressed genes among UC, OSK, and OSKE groups at D3 and D10. Related gene ontology analysis is on the right of the heatmap. **h** Selected marker genes highly activated by OSKE. **i** Expression levels of pluripotent transcription factors SOX2 and OCT4 in OSK and OSKE conditions. **j** Expression levels of endogenous ERVH48-1 (Endo-ERVH48-1) and Total-ERVH48-1 in OSK, OSKE conditions and UCs, iPSCs, and H1

To study the role of ERVH48-1 in the reprogramming process, we collected and isolated two strains of urine cells (UC-P and UC-L) from healthy volunteers. We successfully constructed an overexpression vector, pCEP4-ERVH48-1 (Fig. 2a). Reprogramming experiments were conducted using the OSK plus miR-302–367 cluster system of non-integrated in vitro chromosomal vectors, using pCEP4-GFP of the same mass as pCEP4-ERVH48-1 as a control (Fig. 2b). Over time, the morphology of UCs introduced into different vectors by electroporation changed, as cells gradually aggregated into clusters. By Day 11 distinct peripheral flatter and longer cells were visible, clustering around smaller rounded cells in the center (Fig. 2c). By Day 17, the clones had largely matured and were picked and cultured as stable clones of Urine induced pluripotent stem cells (UiPSCs). The remaining well plates were stained for alkaline phosphatase (AP), and the number of clones was counted to measure reprogramming efficiency (Fig. 2d). The results showed that pCEP4-ERVH48-1 significantly promoted the reprogramming of UCs into UiPSCs (Fig. 2e), suggesting that ERVH48-1 enhances urine cell reprogramming.

### Mechanisms of reprogramming efficiency improvement after addition of ERVH48-1

We performed bulk RNA-seq of urine cells at D3 and D10 during reprogramming. Principal component analysis (PCA) revealed that cells reprogrammed with the addition of ERVH48-1 (OSKE) were slightly faster than normal cells (OSK) at D3, but significantly faster at D10 (Fig. 2f). It is possible that the faster changes are responsible for the increased efficiency. GO analysis revealed that at D3, compared to normal reprogramming, the expression of genes related to DNA repair, telomere organization, and DNA replication pathways was lower in cells reprogramed with the addition of ERVH48-1 (Fig. 2g). Conversely, the expression of genes related to phosphorylation, regulation of TORC1 signaling and lipid transport pathways were more upregulated, consistent with the early stage of reprogramming (Fig. 2g). At D10, compared to regular reprogramming, gene expression changes were related to intracellular signal transduction, phospholipid biosynthetic process, and ubiquitin dependent protein catabolic process pathways was lower in cells reprogramed with addition of ERVH48-1 (Fig. 2g). Meanwhile, gene expression of genes related to nucleosome assembly, positive regulation of transcription by RNA polymerase II, and chromatin organization pathways was upregulated, consistent with a faster cell transformation rate in reprogramming with addition of ERVH48-1 (Fig. 2g).

We then summarized the expression of the three pluripotency genes, LIN28A (Yu et al. 2007), SALL4 (Zhang et al. 2006), and GDF3 (Clark et al. 2004), during reprogramming and the expression of ERVH48-1 itself. We found that, compared to normal reprogramming, the expression of ERVH48-1 in reprogramming with the addition of ERVH48-1 could reach its peak on day 3 and had begun to fall back by day 10, whereas during normal reprogramming, it took until day 10 for ERVH48-1 to peak (Fig. 2h). We observed that all three pluripotency genes, LIN28A, SALL4, and GDF3, showed a significant increase in expression on day 10 in ERVH48-1 reprogramming, much higher than that in normal reprogramming (Fig. 2h). Additionally, during reprogramming with the addition of ERVH48-1, the expression of the exogenously introduced pioneer factors SOX2 and OCT4 were still rising by day 10 (Fig. 2i). This may suggest that the decline in exogenous expression was accompanied by a greater rise in endogenous expression, whereas a decline in SOX2 and OCT4 expression were observed on day 10 of the common reprogramming process (Fig. 2i). In conclusion, these results suggest that ERVH48-1 promotes reprogramming likely by accelerating the process and enhancing the expression of pioneer factors such as OCT4 and SOX2 (Soufi et al. 2015), thereby increasing the efficiency of reprogramming.

Next, we designed a real-time polymerase chain reaction (qPCR) primer to detect both exogenous and endogenous ERVH48-1 by targeting protein code sequence (CDS) (Total-ERVH48-1), and a primer targeting the 3’UTR of endogenous ERVH48-1 (Endo-ERVH48-1) (Fig. 2j). According to our results, endogenous ERVH48-1 was not activated in D3 and D10 but was upregulated at D17 in normal OSK reprogramming system (Fig. 2j). Interestingly, when ERVH48-1 was introduced into the reprogramming system (OSKE), total ERVH48-1 was upregulated on D3 to D17. We observed a slight activation of endogenous ERVH48-1 at D10, with significant activation at D17 (Fig. 2j). At D17, during the late reprogramming stages, total ERVH48-1 levels were comparable to endogenous ERVH48-1 levels in both OSK and OSKE conditions (Fig. 2j), suggesting that exogenous ERVH48-1 was silenced and endogenous ERVH48-1 was activated and maintained until iPSC formation.

### Quality testing of ERVH48-1-generated UiPSCs

**Fig. 3:**
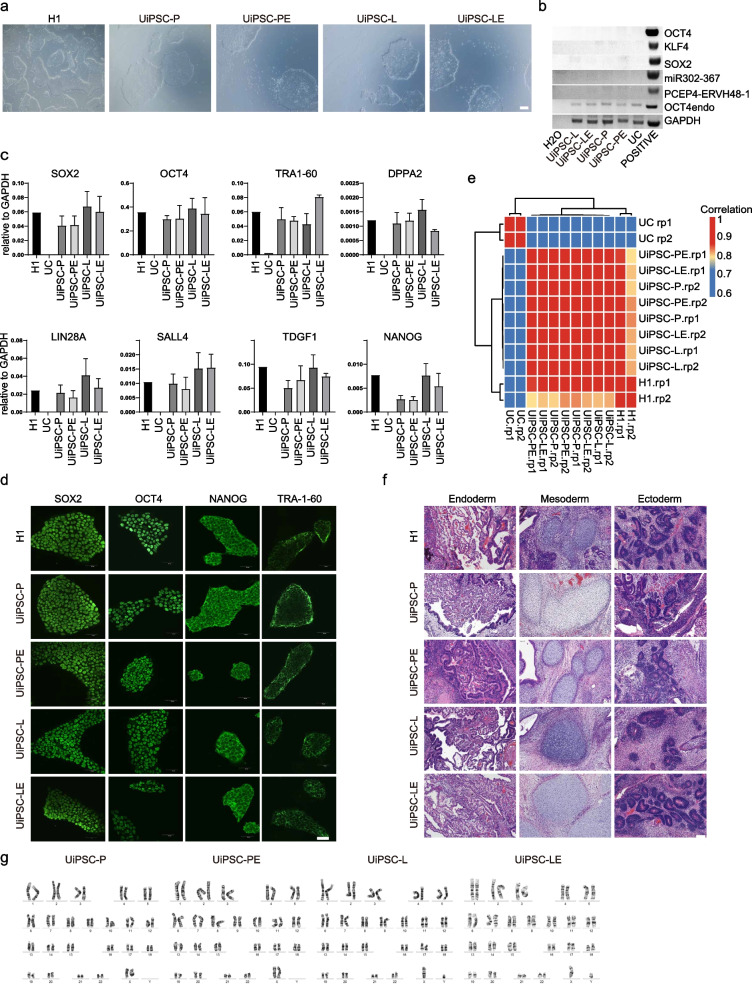
Quality testing of ERVH48-1-generated UiPSCs. **a** The morphology of UiPSCs resembles that of hPSC (scale: 500 μm). **b** Genomic DNA polymerase chain reaction (PCR) assay of UiPSCs cell line to analyze exogenous reprogramming factor integration. **c** qPCR results for pluripotent markers in UiPSCs cell lines. Data were from three independent experiments and are shown as the mean ± SEM. **d** Immunofluorescence images of pluripotent markers (OCT4, SOX2, NANOG, and TRA-1–60) in UiPSCs cell lines (scale: 50 μm). **e** Correlation coefficient matrix between UiPSCs cell line, hPSC, and UCs. **f** Teratoma detection in UiPSCs and hPSCs. HE staining was performed 7 weeks after subcutaneous injection, with histology consistent with endoderm, mesoderm, or ectoderm. (Scale: 100 μm). **g** Karyotypic analysis of these iPSC clones

UiPSCs derived from control induction were labeled as UiPSC-P, UiPSC-L, while those generated with ERVH48-1 were labeled as UiPSC-PE and UiPSC-LE. The cellular morphology of these UiPSCs was consistent with that of the H1 cell line and without integration of the exogenous reprogramming factors (Fig. 3a and b). qPCR assay showed that the expression of pluripotency genes, including SOX2, OCT4, NANOG, LIN28A, SALL4, were similar to those in H1 across all four UiPSC lines (Fig. 3c). Immunofluorescence revealed that the pluripotent markers SOX2, OCT4, TRA-1–60 and NANOG were present in all four UiPSC lines, with cellular localization identical to that of H1 (Fig. 3d). RNA-seq analysis of UiPSC-P, UiPSC-L, UiPSC-PE, UiPSC-LE, H1 and UCs showed strong cross-correlation between UiPSC-P and UiPSC-PE, as well as between UiPSC-L and UiPSC-LE, with overall expression profiles similar to H1 but distinctly different from UCs (Fig. 3e). Furthermore, teratoma experiments indicated that all four cell lines UiPSC-P, UiPSC-L, UiPSC-PE, UiPSC-LE and H1 developed tumours within one month. HE staining of the teratomas confirmed the presence of tissues from all three germ layers in all groups (Fig. 3f). Karyotype analysis of iPSCs generated with and without ERVH48-1 supplementation demonstrated that all clones had a normal karyotype, suggesting that ERVH48-1 does not induce chromosomal abnormalities during reprogramming (Fig. 3g). These findings indicate that UiPSC-P, UiPSC-L, UiPSC-PE, and UiPSC-LE have the same multidirectional differentiation potential as H1.

### UiPSCs can differentiate into neural cells in vitro

**Fig. 4:**
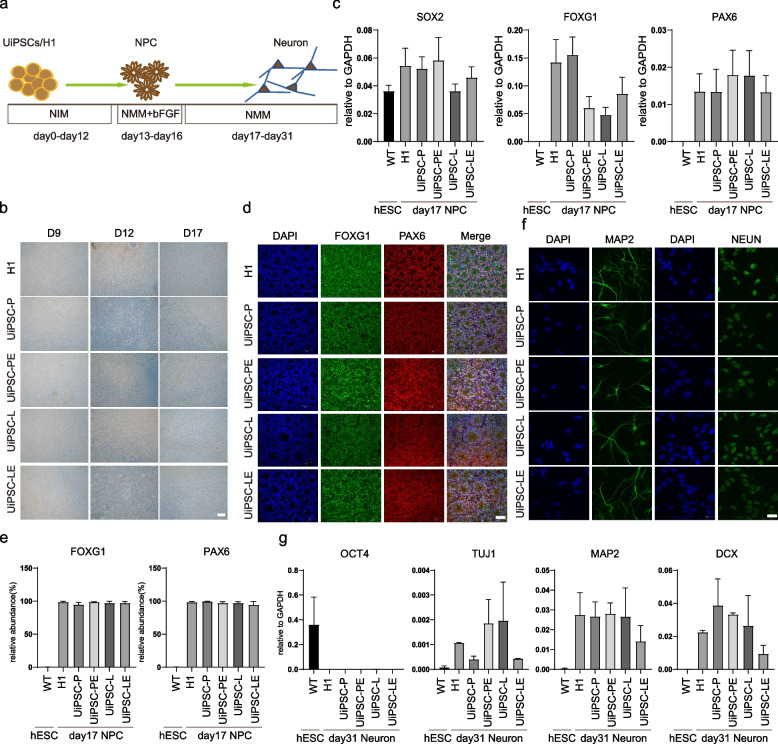
UiPSCs can differentiate into neural cells in vitro. **a** Schematic diagram of the neural differentiation process. **b** Cell morphology at different time points during neuronal differentiation of the cell lines. **c** Relative mRNA expression levels of neural precursor markers (PAX6 and FOXG1) and pluripotency markers (SOX2) in cells on day 17 of differentiation. Data were from three independent experiments and are shown as the mean ± SEM. **d** The protein expression of neural precursor markers PAX6 and FOXG1 was detected by immunofluorescence assay on day 17 of differentiation. Nuclei were stained with DAPI (scale: 50 μm). **e** Flow cytometry performed on day 17 of neuronal differentiation, and the proportion of PAX6^+^ and FOXG1^+^ cells was recorded. Data were from three independent experiments and are shown as the mean ± SEM. **f** Immunofluorescence assay detected the expression of neuronal markers MAP2 and NEUN in the cells on day 31 of differentiation. Nuclei was stained with DAPI (scale: 50 μm). **g** Relative mRNA expression levels of the pluripotency markers (OCT4) and neuronal markers (TUJ1, MAP2, DCX) in cells on day 31 of differentiation. Data were from three independent experiments and are shown as the mean ± SEM

To assess the ectodermal differentiation potential of each iPSC clone, the four cell lines, UiPSC-P, UiPSC-L, UiPSC-PE, and UiPSC-LE, were induced to differentiate into neuronal cells in vitro (Fig. 4a). By day 13 of neural differentiation, cells began forming neural wreaths, progressively differentiated into mature neural precursor cells (NPCs) (Fig. 4b). qPCR analysis revealed that the NPC-specific marker genes FOXG1 and PAX6 were up-regulated in the NPCs derived from UiPSC-P, UiPSC-L, UiPSC-PE, UiPSC-LE, and H1 by day 17, compared to undifferentiated H1 (Fig. 4c). Immunofluorescence confirmed that the induced neural precursor cells expressed the relevant markers FOXG1 and PAX6, and exhibited clear neural rosettes structure (Fig. 4d). Flow cytometry assay showed that more than 80% of cells in all groups were positive for FOXG1 and PAX6 (Fig. 4e). These cells were further differentiated into neurons. By day 24, a network of spindle-shaped and interconnected neuronal cells was visible. On day 31, immunofluorescence assays indicated that the neuronal markers MAP2 and NEUN were expressed in the differentiated neuronal cells across all groups, with microtubule-like neural axons also visible (Fig. 4f). qPCR showed that neuronal cell-specific marker genes TUJ1, DCX, and MAP2 were up-regulated, while the pluripotency gene OCT4 was down-regulated in neuronal cells differentiated from UiPSC-P, UiPSC-L, UiPSC-PE, UiPSC-LE, and H1, compared to undifferentiated H1 (Fig. 4g). The results demonstrate that UiPSCs generated with ERVH48-1 supplementation have the potential to differentiate into neural cells.

### UiPSCs can differentiate into cardiomyocytes in vitro

**Fig. 5:**
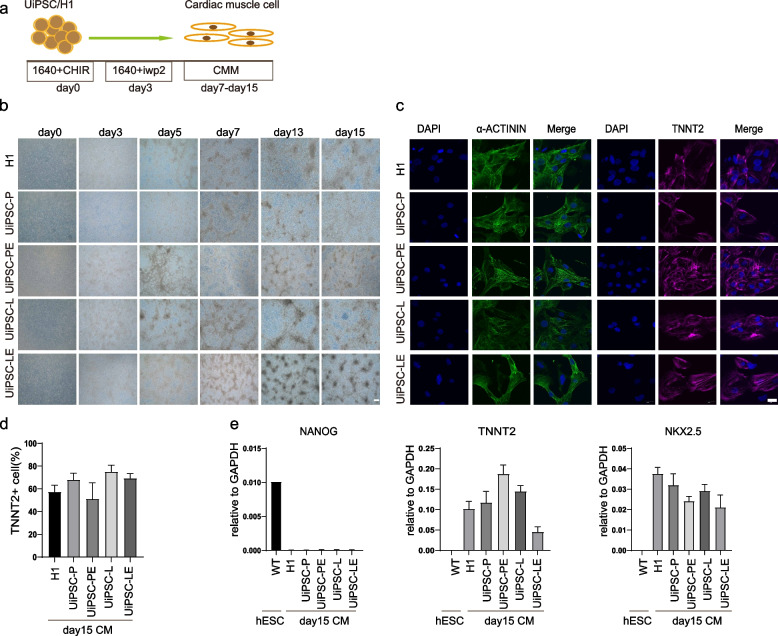
UiPSCs can differentiate into cardiomyocytes in vitro. **a** Schematic diagram of the myocardial differentiation process. **b** Cell morphology at different time points during myocardial differentiation from UiPSCs and hPSCs. **c** Immunofluorescence assay detected the expression of TNNT2 and α-actin in differentiated cardiomyocytes. Nuclei was stained with DAPI (scale: 20 μm). **d** Proportion of TNNT2 + cells after 15 days of cardiomyocyte differentiation was measured by flow cytometry. **e** qPCR detection of the expression of pluripotency marker NANOG and cardiomyocyte markers NKX2-5, TNNT2. Data were from three independent experiments and are shown as the mean ± SEM

To evaluate the mesodermal differentiation potential of UiPSC-P, UiPSC-L, UiPSC-PE, and UiPSC-LE, these cell lines were induced to differentiate into cardiomyocyte in vitro (Fig. 5a). By day 15 of cardiac differentiation, most cells exhibited typical cardiomyocyte morphology and spontaneous contractions were observed (Fig. 5b, Supplementary Material 2, 3, 4, 5, and 6). Immunofluorescence assays confirmed the expression of cardiomyocyte markers TNNT2 and α-ACTININ in the cardiomyocytes derived from UiPSC-P, UiPSC-L, UiPSC-PE, UiPSC-LE, and H1 on day 15 (Fig. 5c). Flow cytometry further revealed that TNNT2 + cells were present in all 4 UiPSCs derived cardiomyocyte groups and H1 (Fig. 5d). qPCR assays demonstrated that the cardiomyocyte-specific marker genes NKX2.5 and TNNT2 were up-regulated, while the pluripotency gene NANOG was down-regulated in the cardiomyocytes differentiated from the 4 UiPSC lines and H1, compared to undifferentiated H1 (Fig. 5e). The results indicate that UiPSCs generated with the addition of ERVH48-1 possess the potential to differentiate into cardiomyocytes.

### UiPSCs can differentiate into lung progenitor cells

**Fig. 6:**
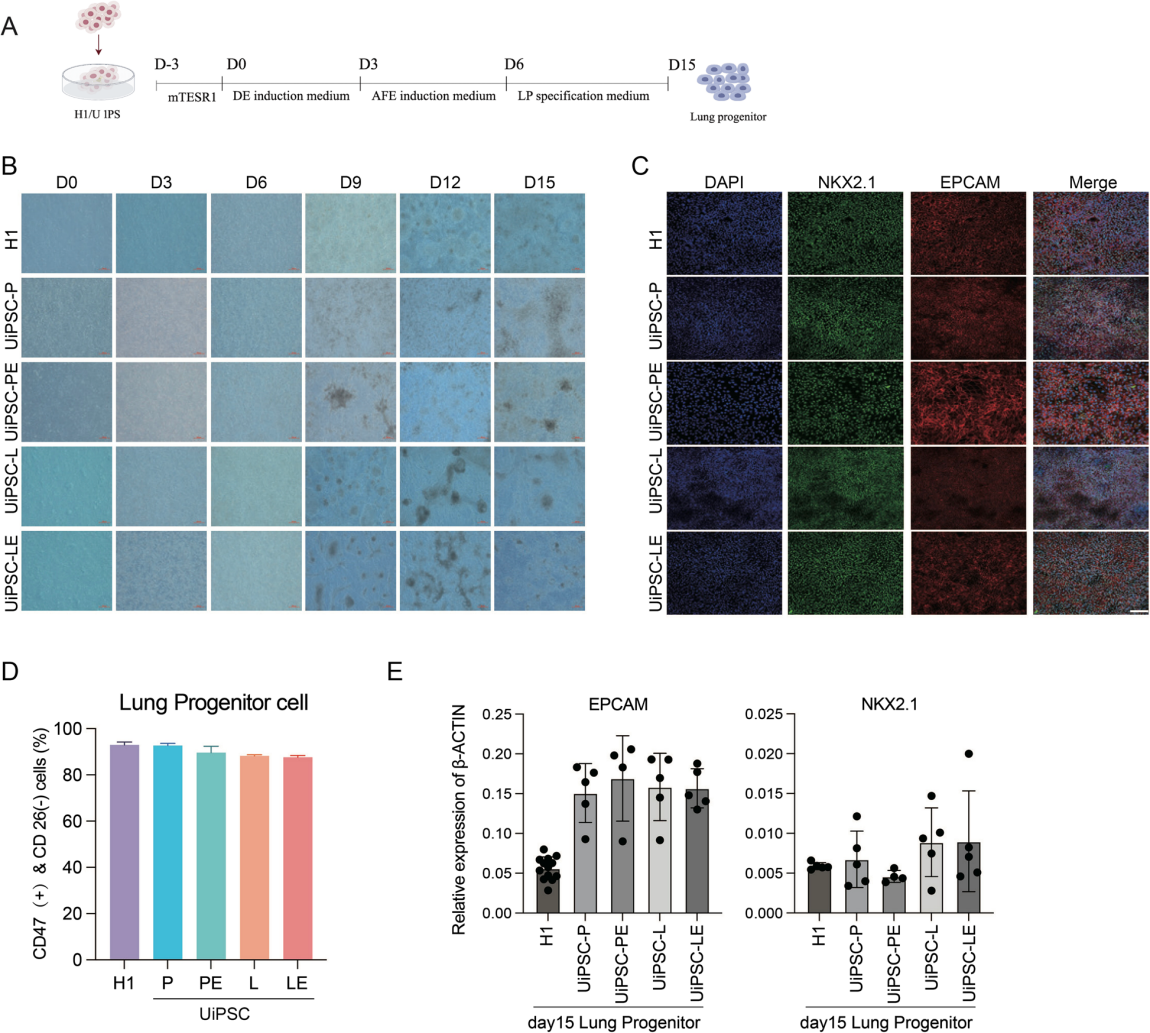
UiPSCs can differentiate into lung progenitor cells in vitro. **a** Schematic diagram of lung progenitor cell differentiation. **b** Cell morphology at different time points during cell line differentiation into lung progenitor cells (scale: 200 μm). **c** Detection of lung progenitor cell markers (NKX2.1 and EPCAM) by immunofluorescence assay on day 15 after differentiation. Nuclear staining with DAPI (scale: 100 μm). **d** Flow cytometry detection of pulmonary progenitor cell markers (CD47 + &CD26- and EPCAM) on day 15 of differentiation. **e** Detection of lung precursor cell markers (NKX2.1 and EPCAM) in cells on day 15 of differentiation by qPCR

To explore the endodermal differentiation potential of UiPSC-P, UiPSC-L, UiPSC-PE, and UiPSC-LE, we induced these UiPSCs to differentiate into lung progenitor cells (LPCs) in vitro (Rodrigues et al. 2021) (Fig. 6a). The cell morphology during differentiation is shown in Fig. 6b. On day 15 of lung progenitor cell differentiation, immunofluorescence assay showed that UiPSC-P, UiPSC-L, UiPSC-PE, UiPSC-LE and H1-induced LPCs expressed lung progenitor cell markers NKX2.1 and EPCAM (Fig. 6c). Flow cytometry showed that the 4 UiPSCs and H1-induced lung progenitors produced CD46 + , with more than 80% of them being CD27- cells (Fig. 6d). qPCR assay showed that the LPCs specific marker genes NKX2.1 and EPCAM were up-regulated in all 4 UiPSCs-derived and H1-differentiated LPCs compared with undifferentiated H1 (Fig. 6e). These results suggest that UiPSCs generated with ERVH48-1 supplementation have the potential to differentiate into LPCs.

## Discussion

Human ERVs (ERVs) are inherited across generations within the host genome and have acted as powerful evolutionary forces, shaping the genomes of higher organisms. Some ERVs retain the ability to induce new integrations into the host genome (Rowe and Trono 2011b). Newly integrated ERVs can regulate the expression of neighboring genes, leading to the emergence of new adaptive genetic variants and resistance to infections in the host. However, they may also have negative consequences, such as spontaneous immunodeficiencies, carcinogenesis, and neurodegenerative changes (Ueda et al. 2020). ERVs exemplify how viruses and their hosts can form mutually beneficial relationships through evolution, advancing our understanding of evolution itself.

ERV insertions and mutations have increased the genetic diversity of the human genome, providing material for natural selection and influencing human evolution in many ways (Chen et al. 2022). For example, syncytins 1 and syncytin 2 play key role in the formation of placental syncytiotrophoblasts and the maintenance of trophoblast cell fusion. The ability of virally encoded proteins to fuse with host cell membranes has been recruited by host cells, facilitating placental invasion into the mother's uterus and preventing maternal immunological rejection of the fetus. thereby offering an evolutionary advantage for embryonic development (Luganini and Gribaudo 2020). Epigenetic regulation mediated by ERVs, particularly those represented by LTR6B, plays a role in human definitive endoderm development (Wu et al. 2022).

ERVH48-1 is a secreted protein with a transmembrane region and binds to the syncytin-1 receptor, ASCT2 to inhibit syncytin-1-mediated cell fusion (Sugimoto et al. 2013). Sugimoto et al. identified a low-activity promoter region within the U3, R, and U5 regions of the 5'LTR sequence of ERVH48-1. In contrast, the EIE sequence, located approximately 2072 bp downstream of the 5'LTR sequence, contains an enhancer region that may bind to GATA and participate in the regulation of placenta-specific expression of ERVH48-1 (Sugimoto et al. 2023). Chen et al. found that ERVH48-1 can act as a competitive endogenous RNA, increasing the expression of WNT2B by adsorbing miR-4784, thereby activating the Wnt/β-catenin signaling pathway. This suggests that ERVH48-1 may be a potential therapeutic target for treatment-resistant prostate cancer (Chen et al. 2023). Additionally, ERVH48-1 has been shown to influence disease outcomes, such as inhibiting breast cancer (Zavesky et al. 2024), prediction of recurrence-free survival in hepatocellular carcinoma (Luo et al. 2020), up-regulation of expression in lung cancer cells (Zhan et al. 2022), and potentially playing a role in antiviral immunity (Frank et al. 2022).

Our recent work provide ERVH48-1 was needed during differentiate hPSCs into cardiomyocytes (Zhang et al. 2024). In this study, explored the role of ERVH48-1 in a somatic cell reprogramming system to uncover the role of ERVH48-1 in pluripotency formation. Reprogramming, the process of reversing cell fate by artificially introducing transcription factors or chemical small molecules, has become an important platform for studying cell fate regulation. As the inverse of embryonic development, reprogramming involves interactions among various factors and proteins, along with accompanying epigenetic phenomena such as DNA methylation, histone modification, and altered chromatin accessibility state (Chen et al. 2013; Li et al. 2021; Sugimoto et al. 2013; Vidal et al. 2020), making it particularly suitable for studying genes related to embryonic development. In our study, we found that ERVH48-1 can promote reprogramming to produce normal iPSCs by active pluripotency gene expression network. Moving forward, we will use reprogramming as a platform to study the effect of ERVH48-1 on cell fate transition to further understand its impact on embryos as well as evolution.

## Materials and methods 

### Collection and expansion of UCs

We enrolled two volunteers who provided urine cells (UCs) following detailed explanations of the study’s aims and the process of UC isolation and induced pluripotent stem cell (iPSC) generation, all of which were approved by the Institutional Review Board (IRB) at Guangdong Provincial People’s Hospital. Any questions raised by the donors were fully addressed. Subsequently, we obtained signed consent forms and collected urine from each participant. The collection of UCs was conducted as previously described (Zhou et al. 2012). In brief, urine samples were collected at the mid-stream from 2 individuals. Each urine specimen was centrifuged at 400 g for 10 min, and the precipitated cells were resuspended by adding RM medium, and cultured in six-well plates packed with 1% gelatin solution (stemcell,07903) for at least half an hour beforehand. RM medium was made by mixing REGM medium (CC-3190, Lonza) with 10% FBS (NTC,SFBE) and DMEM (Gibco, 11,965,092) medium in equal proportions and reconstituted by adding 1 × P/S and 1 μg/ml Promicin (ant-pm-2, Invivogen) when needed. Then, the process was repeated every other day until the urine-derived cells were vigorously expanded.

### Cloning of overexpression vectors

The ERVH48-1 CDS fragment was obtained by PCR using cDNA from human pluripotent stem cells (H1) as a template, recombinantly ligated to the pCEP4 vector backbone digested using NotI and BamHI and transformed, single clones were picked for amplification, and the overexpressed pCEP4-ERVH48-1 plasmid was obtained after PCR identification and sequencing.

### iPSC generation

Reprogramming experiments were conducted following the protocol of Li et al. (Wang et al. 2013). Initially, we digest cells with 0.05% trypsin (Gibco,25,300,054), then counted and collected 600,000 urine cells. Subsequently, reprogramming plasmids were transferred into urine cells using electroporation and inoculated into six-well plates precoated with Matrigel (Corning,354,277). The plasmids combination used in the control group consisted of 3 μg of pCEP4-EO2S-ET2K (including OCT4, SOX2, SV40LT, and KLF4), 3 μg of pCEP4-miR-302–367 cluster (including miR-302b, c, a, d, and miR367), and 3 μg of pCEP4-GFP. In contrast, the experimental group utilized 3 μg of pCEP4-EO2S-ET2K, 3 μg of pCEP4-miR-302–367, and 3 μg of pCEP4-ERVH48-1. The reprogramming process was initiated on day 0 in urine cell culture medium RM. During the early reprogramming stage (day 1 to day 10), 0.5 μM A-83–01.(Sigma,SML0788-5MG), 3 μM CHIR99021 (Guangzhou Laura Biotech,252,917–06–9), 0.5 μM Tzv (Guangzhou Laura Biotech,1,226,056–71–8), 250 μM sodium butyrate (Sigma,303,410-100G), 0.3 μM cyclic pifithrin-a (Stemgent,04–0040) were added to the RM culture medium to facilitate the transition from UCs to UiPSCs. Subsequently, in the later stage (day 11 to day 17), RM was replaced with mTeSR1 medium (stemcell,85,850), and the 5 small molecules along with compound 0.5 μM PD0325901 (merck,391,210–10-9) were added to induce the formation of pluripotent stem cells. By approximately day 17, mature cell clusters appeared on the surface, UiPSC colonies were separated for individual cultivation, and the mTeSR1 medium was changed daily to continuously purify the pure UiPSCs through passaging and clone selection.

### Alkaline phosphatase staining

The AP staining method described by Wang et al. (Wang et al. 2013) was utilized. The six-well plate was first cleaned with PBS, followed by the addition of 1ml of 4% PFA at room temperature for 10 min. After rinsing with PBS three times, 2.5ml of the prepared working solution (Beyotime,C3206) was added and incubated at room temperature for 20–30 min in the dark. Subsequently, the plate was washed with PBS three times, images were taken to document the AP staining, and the number of clones was counted.

## PCR analysis

We employed the PCR analysis method for exogenous reprogramming factors and exogenous trunk integration genes as described by Xue et al. (Xue et al. 2013). The PCR process was carried out using Phanta enzyme (5022, Vazyme). The primers used in this study are listed in Table S1.

### qPCR analysis

ChamQ SYBR qPCR Master Mix (Q711-02, Vazyme) was utilized for the qPCR reaction. All qPCR primers mentioned in this paper can be found in Table S1.

### Immunofluorescence

The cells were fixed in 4% PFA at room temperature for 15 min and per meabilized with 3% fetal bovine serum containing 0.25% Triton-X100 at room temperature for 40 min. Subsequently, the cells were incubated overnight at 4℃ with the primary antibody, followed by incubation with a suitable second ary antibody the next day. Both primary and secondary antibodies were diluted in PBS with 3% fetal bovine serum. The nuclei were stained with DAPI (Bey otime, C1002) and visualized using a confocal microscope (Olympus 3000). The antibodies utilized in this study are listed below: OCT4 (abcam, ab181557), SOX2 (R&D, AF2018), NANOG (abcam, ab21624), TRA-1–60 (abcam, ab1628.8), PAX6 (Covance**,** PRB-278P), FOXG1 (abcam, ab18259), MAP2 (invitrogen, 13–1500), NEUN (abcam, ab177487), TNNT2 (BD Pharmingen, 565,744), α-acti nin (CST, 6487S), Goat anti-Mouse Alexa Fluor™ 568 (invitrogen, A-11004), Goat anti-Rabbit Alexa Fluor™ 568 (invitrogen, A-11011), Goat anti-Rabbit Al exa Fluor™ 488 (invitrogen, A-11008), Donkey anti-Goat Alexa Fluor™ 568 (i nvitrogen, A-11057), Donkey anti-Goa Alexa Fluor™ 647 (invitrogen, A-21447),

NKX2.1 (abcam, ab76013), EpCAM (abcam, ab7504), APC anti-human CD47 (Biolegend, 323,124), PE anti-human CD26, (Biolegend, 302,706).

### Flow cytometry analysis

The cells were fixed in 4% paraformaldehyde at room temperature for 15 min, washed with PBS, and then resuspended in PBS with 0.25% TritonX-100 and 3% fetal bovine serum. After a 40-min incubation at room temperature, the cells were sequentially incubated with the appropriate primary and secondary antibodies. Flow cytometry data were acquired using a BD flow analyzer and analyzed with FlowJo software.

### Teratoma determination

The differentiation potential of human induced pluripotent stem cells in vivo was assessed using the teratoma method. Immunodeficient NOD/SCID/IL2rg − / − (NSI) mice, lacking mature T cells, B cells, and natural killer cells, were used to facilitate transplantation of a wide range of primary human cells. Induced pluripotent stem cells were collected, suspended with matrigel, and subcutaneously injected into 6-week-old immunodeficient NSI mice. Teratomas were obtained in all injection groups after 4 weeks. The teratomas were collected at 7 weeks, fixed with 4% paraformaldehyde, and subjected to H&E staining in the GIBH Laboratory Pathology Department.

### Cell culture

Human embryonic stem cell (H1) cell lines were obtained from WiCell (WA01/H1; WiCell, USA). The human UiPSCs cell line used in this study was derived from urine from a healthy donor. H1 and human UiPSCs were cultured in mTeSR1 medium on matrigel-coated plates. When the cell density reached 50–60%, Accutase (Stemcell, 07920) was used for digestion at 37 °C for 5 min, followed by incubation in mTeSR1 medium supplemented with 5 μM ROCK inhibitor Y-27632 (Selleck,S1049) for 24 h. Subsequently, the culture medium was changed to mTeSR1 medium without Y27632.

### Myocardial differentiation of human ESCs and human UiPSCs cell lines

Cardiac differentiation was performed according to the RPMI differentiation protocol referenced in zhong et al. (Zhong et al. 2023). H1 and UiPSCs were dissociated using Accutase at 37 °C for 5 min, and then 250,000 cells per well were seeded in a sixwell plate. The cells were cultured in mTeSR1 for 2–3 days with daily medium changes until reaching a cell density of approximately 70%, designated as day 0. On day 0, the cells were treated with RPMI medium (Gibco, C11875500BT) containing 6 μM CHIR990211 (Selleck, S2924) for 24 h, followed by removal of CHIR. On day 5, fresh RPMI medium containing 2.5 μM IWP2 (Tocris,3533) was added. Starting from day 7, the cells were maintained in cardiomyocyte maintenance medium (CMM: RPMI supplemented with ITS-A supplement (100X) (Gibco,51,300,044) and 200 μg/ml ascorbate 2-phosphate magnesium salt hydrate (Sigma,A8960), with medium changes every 2 days. Cardiomyocytes that had differentiated and matured by day 15 were harvested for analysis.

### Neuron differentiation of human ESCs and human UiPSCs

Neuronal differentiation of UiPSC cell lines via neural precursor cells was conducted following the protocol proposed by Shi et al. (Shi et al. 2012). Initially, 1 million cells per well were seeded in a 12-well plate on day 0. During the early stage (day 1 to day 11), The neural differentiation induction medium (NIM) supplemented with 10 μM SB431542 (Selleck, S1067) and 1 μM Dorsomorphin (Tocris, 3093) in neural maintenance medium (NMM: 24 ml DMEM/F12(Gibco, 11,320,033), 24 ml Neurobasal (Gibco, 21,103,049), 0.5 × B-27 (Gibco, 17,504,044), 0.5 × N2 (Gibco, 17,502,048), 0.5 × GlutaMax (Gibco, 35,050–061), 1 × MEM NEAA (Gibco, 11,140,050), 0.5 mM sodium pyruvate (Gibco, 11,360,070), 50 U/ml penicillin / 50 μg/ml streptomycin.(Gibco, 15,070,063), 5 μg/ml insulin (Sigma, I9278), 50 μM 2-mercaptoethanol (gibco, 21,985,023) was changed daily. On the 12th day, the cells were dissociated for 5 min with dispase (Life Technologies, 17,105), and then mechanically dissociated into small clumps. The cells were centrifuged, resuspended in NIM medium supplemented with 5 μM ROCK inhibitor Y-27632, and plated in a polylysine (sigma, P4957) and laminin (sigma, L2020)-coated six-well plate. Subsequently, NMM neural maintenance medium with 20 ng/ml bFGF (PeproTech, 100-18B) was replaced daily from day 13 to day 16. On day 17, similar methods were used to dissociate the cell clumps and passage approximately 1/3 of them into a new six-well plate. The cells were counted using Accutase on day 24, with around 200,000 cells seeded into new wells. From day 18 onwards, the cells were maintained in NMM neural maintenance medium until neuronal maturation. Neural precursor cells that differentiated on day 17 and cardiomyocytes that matured by day 31 were collected for analysis.

### Differentiation of hPSC into lung progenitor

For lung progenitor differentiation,we modified protocol from previously reported protocol (Rodrigues Toste de Carvalho AL, et al. Nat Protoc. 2021). All hPSC lines were cultured in mTeSR till 70% to 80% confluence, followed by differentiation in serum-free differentiation (SFD) medium composed of DMEM/F12 (3:1) supplemented with 1 × N2, 1 × B27, 1 × Glutamax, 50 μg/ml L-Ascorbic acid (Sigma Aldrich, A4544), 0.4 μM monothioglycerol (Sigma-Aldrich, M6145), 1% penicillin– streptomycin and 0.05% BSA. Briefly, All hPSC lines were induced to differentiate to definitive endoderm (DE) by treatment with 100 ng/ml Activin A (MCE, HY-P70311), 2.5 ng/ml human bFGF, 0.5 ng/ml human BMP4 (Peprotech, 120–05) and 10 μM Y27632 for 3 days in SFD medium. For induction of anterior foregut endoderm, cells were cultured in SFD medium supplement with 1.5 μM Dorsomorphin dihydrochlorid (MCE, HY-13418) and 10μm SB431542 for 24 h and then switched for 24 h to 10 μM. SB431542 and 1 μM IWP2 (Selleck, S7085) treatment. The cells were then cultured for 10 days in SFD medium containing 3 μM CHIR99021, 10 ng/ml human FGF10 (Peprotech, 345-FG-250), 10 ng/ ml human FGF7 (Novoprotein, CM88), 10 ng/ ml human BMP4 and 50 nM all-trans retinoic acid (Sigma-Aldrich, R2625) to promote differentiation into NKX2.1^+^ lung progenitors.

### Bulk RNA-seq and analysis

The raw bulk RNA-seq data of H1 and UCs were downloaded from GSE168392, while the reprogramming cells’ bulk RNA-seq data were generated in this study. Briefly, all sequencing data were aligned to the reference hg38 genome using Bowtie2. Quality control and filtering were performed using Samtools, which included removing lowquality reads, deduplicating reads, and removing ribosomal sequences. Transcripts were quantified using RSEM. Selecting higher expressed gene in H1 and UCs with Log2 (Fold Change) > 1. GO analysis was performed using DAVID to explore the biological pathways involved in differentially expressed genes. The reprogramming path way was analyzed using Principal component analysis (PCA).

### Single-cell RNA-seq (scRNA-seq) data analysis

The raw single cell RNA-seq data of H1 and UCs were downloaded from GSA: HRA003227 and GSE157640. Briefly, the fastq reads are aligned to the transcriptome generated using the hg38 genome and GENCODE annotations with STAR. The filtered matrix was processed using scanpy to exclude low-count cells and cells with high mitochondrial counts. The filtered dataset is subjected to t-distributed stochastic neighbor embedding (t-SNE), with the first two components used for subsequent analysis, such as cell clustering and annotation. The expression of selected marker genes was shown by t-SNE plot and violin plot.

### Statistical analysis

The data are presented as mean ± SEM of at least three independent experiments. Statistical significance was determined using t-tests (*, *p* ≤ 0.05; **, *p* ≤ 0.01; and ***, *p* ≤ 0.001, considered significant).

## Supplementary Information

Supplementary Material 1. Table S1: qPCR primer.

Supplementary Material 2. The beating of cardiomyocytes was induced by H1.

Supplementary Material 3. The beating of cardiomyocytes was induced by UiPSCc-L.

Supplementary Material 4. The beating of cardiomyocytes was induced by UiPSCs-LE.

Supplementary Material 5. The beating of cardiomyocytes was induced by UiPSCs-P.

Supplementary Material 6. The beating of cardiomyocytes was induced by UiPSCs-PE.

## Data Availability

The raw sequence data of this project reported in this paper has been deposited in the Genome Sequence Archive (Genomics, Proteomics & Bioinformatics 2021), China National Center for Bioinformation / Beijing Institute of Genomics, Chinese Academy of Sciences (GSA: HRA007274, HRA008256) and is publicly accessible at https://ngdc.cncb.ac.cn/gsa.

## Abbreviations

ERVs: Endogenous retroviruses
ERVH48-1: Human endogenous retrovirus group 48 member 1
iPSCs: Induced pluripotent stem cells
LTR: Long Terminal Repeat
PBS: Primer Binding Site
PPT: Primer Primer Target
hPSCs: Human pluripotent stem cells
UCs: Urine cells
UiPSCs: Urine induced pluripotent stem cells
AP: Alkaline Phosphatase
PCA: Principal component analysis
OSK: OCT4-SOX2-KLF4
OSKE: OCT4-SOX2-KLF4-ERVH48-1
qPCR: Real-time polymerase chain reaction
PCR: Polymerase chain reaction
NPCs: Neural precursor cells
LPCs: Lung Progenitor Cells
SOX2: SRY-box containing gene 2
OCT4 (POU5F1): Octamer Binding Transcription Factor 4
NANOG: Recombinant NANOG Homeobox Protein
LIN28A: Lin-28 Homolog A
SALL4: Spalt-like transcription factor 4
TRA-1–60: Podocalyxin-like
FOXG1: Forkhead Box G1
PAX6: Paired box protein Pax-6
MAP2: Microtubule-associated protein 2
NeuN: Neuronal Nuclei Antigen
TUJ1: β-Tubulin
DCX: Doublecortin
MAP2: Microtubule Associated Protein 2
NKX2.5: NK2 homeobox 5
TNNT2: Cardiac troponinT 2
NKX2.1: NK2 homeobox 1
ECPAM: Endocrine and Exocrine Pancreatic Antigen Marker
ASCT2: Solute Carrier Family 2 Member 2
