# Supplementary material for: Endogenous retroviral ERVH48-1 promotes human urine cell reprogramming
Source: Cell Regen. 2024 Sep 13;13:17. doi: 10.1186/s13619-024-00200-2 (PMC11399365; doi:10.1186/s13619-024-00200-2)
Supplement: Supplementary file 1 — Supplementary Material 1. Table S1: qPCR primer. [file 13619_2024_200_MOESM1_ESM.pdf]

Supplementary Information

Table S1:

qPCR primer		
name	Forward primer	Reverse primer
SOX2	CCCAGCAGACTTCACATGT	CCTCCCATTTCCCTCGTTTT
OCT4	CCTCACTTCACTGCACTGTA	CAGGTTTTCTTTCCCTAGCT
NANOG	TGAACCTCAGCTACAAACAG	TGGTGGTAGGAAGAGTAAAG
TRA-1-60	TCATCATCACCATCGTCTG	GTAACCATTCTCCACTGTCT
SALL4	ATCCACCTGTCCTCATCA	GGGCTGTCTCTGTCTTTAG
LIN28A	CCTGGTGGAGTATTCTGTATT	ATATGGCTGATGCTCTGG
DPPA2	TGGATAGCAGCAAGAAGAAT	AAGTAGATGACCTGGATTGTATT
TDGF1	CTTCTACGGACGGAACTG	TGCTCATCCATCACAAGG
GDF3	GTGGCAGAGGTTCTTACAA	TCCTGGAGATACTGGTCAA
GAPDH	GTGGACCTGACCTGCCGTCT	GGAGGAGTGGGTGTCGCTGT
TNNT2	TTCACCAAAGATCTGCTCCTCGCT	TTATTACTGGTGTGGAGTGGGTGTGG
NKX2.5	CAAGTGTGCGTCTGCCTTT	CAGCTCTTTCTTTTCGGCTCTA
PAX6	AACGATAACATACCAAGCGTGT	GGTCTGCCCGTTCAACATC
FOXG1	GAGCGACGACGTGTTCATC	GCCGTTGTAACTCAAAGTGCTG
TUJ1	CCCGTTATCCCAGCTCCAATATGCT	ATGGCTTGACGTGCGTACTTCTCC
MAP2	AAACTGCTCTTCCGCTCAGACACC	GTTCACTTGGGCAGGTCTCCACAA
DCX	CTCTGGATGAAAATGAATGCCGA	AGGGCTCTTGGCTGAAGTCT
NKX2.1-1	AGCACACGACTCCGTTCTC	GCCCACTTTCTTGTAGCTTTCC
β-actin	TTTGAATGATGAGCCTTCGTGCCC	GGTCTCAAGTCAGTGTACAGGTAAGC
ECPAM	CCATGTGCTGGTGTGTGAAC	CCTTCTGAAGTGCAGTCCGC
Total-ERVH48-1	GCACTACAGAGGGGAGATGC	CCCCATTACGACTGCCACAT
Endo-ERVH48-1	CAGGTGCTTGAGGACATA	ATTGGACACATTCATGGTAAG
PCR primer		
name	Forward primer	Reverse primer
OCT4	AGTGAGAGGCAACCTGGAGA	AGGAACTGCTTCCTTCACGA
KLF4	CCCACACAGGTGAGAAACCT	CCCCCTGAACCTGAAACATA
SOX2	ACCAGCTCGCAGACCTACAT	CCCCCTGAACCTGAAACATA
miRNA	TTTCCAAAATGTCGTAATAACCCCG	TCCATGTTTTGGTGATGGTA
PCEP4-ERVH48-1	TTTCCAAAATGTCGTAATAACCCCG	TGATTCAACACATTCCTCGA
OCT4 endo	AGTTTGTGCCAGGGTTTTTG	ACTTCACCTTCCCTCCAACC
GAPDH	ACCACAGTCCATGCCATCA	TGAGCTTGACAAAGTGGTCG

Video S1 The beating of cardiomyocytes was induced by H1.

Video S2 The beating of cardiomyocytes was induced by UiPSCc-L.

Video S3 The beating of cardiomyocytes was induced by UiPSCs-LE.

Video S4 The beating of cardiomyocytes was induced by UiPSCs-P.

Video S5 The beating of cardiomyocytes was induced by UiPSCs-PE.
